# HDL and LDL have distinct, opposing effects on LPS-induced brain inflammation

**DOI:** 10.1186/s12944-023-01817-z

**Published:** 2023-04-24

**Authors:** Daniel E. Radford-Smith, Abi G. Yates, Laila Rizvi, Daniel C. Anthony, Fay Probert

**Affiliations:** 1 Department of Pharmacology, Medical Sciences Division, University of Oxford, Oxford, UK; 2 Department of Chemistry, University of Oxford, Oxford, UK

**Keywords:** Lipoproteins, LPS, Acute phase response, Neuroinflammation, NMR, Metabolomics

## Abstract

Endotoxemia and sepsis induce neuroinflammation and increase the risk of neurodegenerative disorders although the mechanism by which peripheral infection leads to brain inflammation is not well understood. While circulating serum lipoproteins are known immunometabolites with the potential to modulate the acute phase response and cross the blood brain barrier, their contribution to neuroinflammation during systemic infection is unknown. The objective of this study was to elucidate the mechanisms by which lipoprotein subclasses modulate lipopolysaccharide (LPS)-induced neuroinflammation. Adult C57BL/6 mice were divided into 6 treatment groups, including a sterile saline vehicle control group (n = 9), an LPS group (n = 11), a premixed LPS + HDL group (n = 6), a premixed LPS + LDL group (n = 5), a HDL only group (n = 6) and an LDL only group (n = 3). In all cases injections were administered intraperitoneally. LPS was administered at 0.5 mg/kg, and lipoproteins were administered at 20 mg/kg. Behavioural testing and tissue collection was performed 6 h post-injection. The magnitude of peripheral and central inflammation was determined by qPCR of pro-inflammatory genes in fresh liver and brain. Metabolite profiles of liver, plasma and brain were determined by ^1^H NMR. Endotoxin concentration in the brain was measured by the Limulus Amoebocyte Lysate (LAL) assay. Co-administration of LPS + HDL exacerbated both peripheral and central inflammation, whilst LPS + LDL attenuated this inflammation. Metabolomic analysis identified several metabolites significantly associated with LPS-induced inflammation, which were partially rescued by LDL, but not HDL. Endotoxin was detected at significantly greater concentrations in the brains of animals that received LPS + HDL compared to LPS + saline, but not those that received LPS + LDL. These results suggest that HDL may promote neuroinflammation through direct shuttling of endotoxin to the brain. In contrast, LDL was shown to have anti-neuroinflammatory properties in this study. Our results indicate that lipoproteins may be useful targets in neuroinflammation and neurodegeneration associated with endotoxemia and sepsis.

**Supplementary Information:**

The online version contains supplementary material available at 10.1186/s12944-023-01817-z.

## Introduction

Lipoproteins are a heterogeneous population of lipid and protein complexes, typically composed of a central hydrophobic core containing highly insoluble esters and triglycerides [38]. This is surrounded by free cholesterol and an outer configuration of phospholipid and apolipoproteins, the latter playing a crucial role in lipoprotein assembly, transport, and metabolism [15]. While their primary function is the trafficking of cholesterol and other lipid-rich molecules between the liver and other tissues, the involvement of lipoproteins in immune regulation is established [19, 56]. Only recently, however, is their complexity and functional diversity being realised. Lipoprotein-bacterial lipopolysaccharide (LPS) interaction may provide a link between peripheral infection, endotoxemia, and neuroinflammation.

Plasma lipoproteins are classified according to their relative density, which broadly reflects their complementary physiological roles in the absorption and transport of dietary lipids. Low density lipoprotein (LDL) is cholesterol-rich and derived from the depletion of triglycerides by muscle and adipose tissue. Increased levels and retention time of LDL in the plasma are major risk-factors for cardiovascular disease (CVD) [21] and metabolic syndrome [23]. In comparison, nascent high density lipoprotein (HDL) is relatively lipid-poor, acquiring peripheral cholesterol for metabolism by the liver. Because of their important yet contrasting roles in lipid metabolism, HDL is generally branded as ‘good’ cholesterol, while elevated plasma LDL levels are considered ‘bad’. Indeed, the increased consumption of fatty foods as part of a Western-style diet in the last 50 years [26] has led to intense focus on HDL as a protective factor against CVD [16] by reducing peripheral cholesterol levels, and against sepsis, likely by ApoA-I-dependent clearance of LPS via the liver [6, 33]. Reconstituted HDL, a synthetic ApoA-I mimic, is regarded as a promising therapeutic target in a number of diseases, including CVD and sepsis [7, 61]. LDL, conversely, is typically regarded as pro-atherogenic, with statins, LDL-lowering drugs, being a key pharmacotherapy for CVD [53].

The complex and heterogenous protein content of human plasma HDL denotes the significant likelihood of HDL having a much wider scope of involvement in human metabolism than currently appreciated. Indeed, proteomic analysis of normal human serum has revealed the presence of greater than 200 HDL-related proteins, with only a small fraction thought to participate in lipid metabolism [52]. Within the HDL proteome, a striking number of proteins are known to be involved in inflammation and regulation of the immune response. In particular, there appears to be an important modulatory role of HDL in innate immunity and endotoxemia [37], though the underlying mechanisms remain poorly understood.

As part of the acute phase response (APR), which occurs early on during the innate immune response to peripheral infection, HDL undergoes significant remodelling. For example, during the APR there is incorporation of APR-associated proteins including Serum Amyloid A (SAA) and LPS-binding protein (LBP) [30], the former displacing ApoA-I to become the predominant apolipoprotein associated with HDL [4, 8, 25, 52]. This process depletes the ability of HDL to neutralise endotoxin-mediated inflammation [39], as this is largely dependent on ApoA-I [11]. As such, the protective, immunomodulatory benefits of HDL are lost. Instead, HDL and LBP facilitate the presentation of LPS to CD14 on roaming macrophages. The subsequent interaction between LBP, CD14, and toll-like receptor 4 (TLR4) elicits macrophage activation and a robust inflammatory response [27].

Clear evidence in rodents supports the association between LPS-induced systemic inflammation and TLR4-mediated microglial activation in the central nervous system (CNS). Moreover, administration of LPS to humans induces sickness behaviour and robust microglial activation [50]. Despite this, the precise mechanism(s) by which this occurs is not known [24]. In vitro studies have demonstrated that LPS can directly activate microglia through TLR4 stimulation, which leads to neuronal loss through inducible nitric oxide synthase (iNOS) production [28]. Additionally, it has been suggested that LPS may infiltrate brain tissue in an HDL-dependent manner [58]. As almost all LPS exists in complex with HDL in plasma [56], it is possible that HDL, readily able to cross the blood brain barrier (BBB) [14], plays a role in LPS-mediated neuroinflammation. Here we substantiate recent evidence for the role of HDL in mediating LPS transport into the brain, and further provide a link between endotoxemia, lipoproteins, and brain inflammation.

Less is known about the role of native LDL in immune homeostasis and response to infection. Whereas oxidised LDL is recognised by macrophages with proatherogenic and proinflammatory consequences, the immunomodulatory properties of non-oxidised LDL are not understood [40]. LDL is known to be able to promote the clearance of LPS via LDL receptors expressed in the liver [34]. We therefore hypothesize that LDL, in contrast to HDL, plays a minimal or therapeutic role on LPS-induced peripheral and central inflammation.

The present study sought to elucidate the mechanisms by which lipoproteins, in particular HDL and LDL, modulate LPS-induced inflammation and metabolism in the brain, liver, and plasma. We used gene expression and endotoxin assays to determine how HDL and LDL differentially affected peripheral and central immune responses to LPS. The co-administration of HDL with LPS significantly increased inflammation in both the liver and prefrontal cortex (PFC). In contrast, co-administration of LDL with LPS resulted in a significant reduction in inflammatory cytokines. NMR metabolomics showed marked changes to the concentration of immune-related metabolites as a consequence of LPS treatment in plasma, liver, and brain, while distinct metabolic signatures for LPS co-administration with HDL or LDL were also observed. This study reinforces the functional diversity of lipoproteins with distinct inflammatory effects across tissues and lipoprotein classes, and provides a putative role for HDL in the link between peripheral endotoxemia and neuroinflammation.

## Materials and methods

***Animals.*** Male C57BL/6 mice, 8 weeks old, were purchased from Charles River and used in this study. Animals were housed under standard diurnal lighting conditions with *ad libitum* access to food and water. All procedures were carried out in accordance with the UK Animals (Scientific Procedures) Act (1986), and licenced protocols were approved by local committees (LERP and ACER, University of Oxford), carried out under license number P996B4AE.

***Peripheral challenge.*** Animals received an intraperitoneal injection of either sterile saline (n = 9), LPS (0.5 mg/kg, n = 11), or LPS + lipoprotein (derived from human plasma; Lee BioSolutions, US), which was mixed immediately prior to administration. The dose and timepoint (6 h) of the LPS challenge was chosen in accordance with our previous study validating its effect on increasing sickness behaviour, as well as peripheral and central inflammation [9]. LPS was either mixed with HDL (n = 6) at a dose of (20 mg/kg), or LDL (n = 5) at (20 mg/kg) for comparable cholesterol levels. Separately, animals were intraperitoneally injected with HDL (n = 6) or LDL (n = 3) alone, to control for any cross-species immunogenicity of the human lipoproteins in mice.

***Behaviour.*** Mice underwent an open field paradigm as previously described [22] to investigate how HDL and LDL moderate LPS-induced sickness behaviour in mice. 6 h post-injection, mice were placed into the top-left corner of the open field and allowed to explore for 5 min. The number of grid crossings and number of rears were scored by an observer blinded to treatment groups.

***Tissue collection.*** Animal tissue was collected immediately following completion of the open field test. Mice were anaesthetised with 4% isoflurane and blood was collected by cardiac puncture with a heparinised 21G needle. Blood was transferred into a heparinised tube and left at room temperature for 30 min, after which it was centrifuged at 1,300x*g* for 10 min at 4 °C. The supernatant was aspirated, aliquoted, and snap frozen on dry ice. Intracardiac perfusion was then performed using heparinised saline, and fresh liver and whole brain was extracted and snap frozen on dry ice.

***Immunodot blot.*** Lipoproteins were diluted in PBS and transferred onto a nitrocellulose membrane. The membrane was blocked with 5% bovine serum albumin (BSA) in 0.1% PBST for 1 h at room temperature, before being incubated with primary antibodies overnight at 4 °C (HDL APO-A I, 1:5000; LDL APO-B, 1:10,000; EVs TSG101, 1:5000). Detection was performed using appropriate horseradish peroxidase (HRP) conjugated secondary antibodies and visualised with Image J software.

***RNA extraction and cDNA synthesis.*** RNA extraction was carried out on the snap frozen liver and brain tissue using the Qiagen RNEasy Mini Kit (Qiagen Ltd, Manchester, UK) as per the manufacturer’s instructions. Total RNA and RNA purity was measured using a NanoDrop 1000 (Thermo Fisher, UK) and was deemed suitable for cDNA conversion if the 260/280nm ratio (indicating the extent of genomic DNA contamination) was > 2, and 260/230 ratio (indicating phenol contamination) was between 1.8 and 2.2. 1000ng of RNA was then converted to cDNA using the high capacity cDNA kit (Applied Biosystems, Warrington, UK), as per the manufacturer’s instructions.

***Quantitative PCR.*** Real-time quantitative PCR was performed on 25ng of cDNA from liver and brain tissue, in duplicate, using the Roche LightCycler 480 (Roche Life Science, Basel, Switzerland) and SYBR green qPCR master mix (Primer Design, Southampton, UK). Forward and reverse primers for *tnf* (F: GCCTCCCTCTCATCAGTTCTAT, R: TTTGCTACGACGTGG GCTA), *il1b* (F: CAACCAACAAGTGATATTCTCCAT, R: GGGTGTGCCGTCTTTCATTA), *cxcl1* (F: GCTGGGATTCACCTCAAGAAC, TGTGGCTATGACTTCGGTTTG) *ccl2* (F: GAGAGGCTGAGACTAACCCAGA, R: ATCACAGCTTCTTTGGGACACT) were used (Primer Design, Southampton, UK). Data were analysed using the 2^−ΔΔCt^ method and are shown as fold change over controls. Expression levels were normalized using the GAPDH housekeeping gene.

***NMR sample preparation.*** Hepatic and brain metabolites were extracted from samples as previously described [43]. Approximately 100 mg of fresh liver sample or one brain hemisphere was homogenised with a pestle and mortar on dry ice, and further homogenised in 50% acetonitrile (Sigma; v/v) in distilled water by vortex. Samples were centrifuged at 5,000x*g* for 5 min at 4 °C. The supernatants were collected, lyophilised, and stored at -80 °C until NMR analysis. Metabolites were resuspended in 600µL of NMR buffer (75mM sodium phosphate buffer prepared in D_2_O, pH 7.4). Samples were centrifuged at 2,500x*g* for 5 min at 4 °C to remove any particulate matter, before being transferred to a 5 mm NMR tube. For the preparation of plasma for NMR spectroscopy, 75µL of platelet-free plasma was combined with 475µL of NMR buffer and transferred to a 5-mm NMR tube.

***NMR spectroscopy.*** NMR spectra were acquired for each sample using a 700-MHz Bruker AVII spectrometer operating at 16.4T equipped with a ^1^ H (^13^ C/^15^ N) TCI cryoprobe. Sample temperature was stable at 310 K. ^1^ H NMR spectra were acquired as described previously [43] using a 1D NOESY pre-saturation scheme for attenuation of the water resonance with a 2s pre-saturation. For liver samples, an addition pulse sequence, the Wasted-II sequence, was applied with 32 data collections, an acquisition time of 1.5s, a relaxation delay of 2s, and an inter-pulse delay of 287µs. For plasma, a spin-echo Carr-Purcell-Meiboom-Gill (CPMG) sequence was used under the same conditions as Wasted-II, but with a longer pulse interval of 400µs. A fixed receiver gain was used to supress broad signals arising from large molecular weight components such as proteins.

***NMR data pre-processing.*** Resulting free induction decays (FIDs) were zero-filled by a factor of 2 and multiplied by an exponential function corresponding to 0.30 Hz line broadening prior to Fourier transformation. All spectra were phased, baseline corrected (using a 3rd degree polynomial), and chemical shifts referenced to the lactate-CH3 doublet resonance at δ = 1.33 ppm in Topspin 2.1 (Bruker, Germany). Spectra were visually examined for errors in baseline correction, referencing, spectral distortion, or contamination and then exported to ACD/Labs Spectrus Processor Academic Edition 12.01 (Advanced Chemistry Development, Inc.). Noise/baseline regions were identified and discounted from further analyses. The region of the spectra between 0.86 and 8.00 ppm was then divided in to 0.02 ppm width buckets and the absolute value of the integral of each spectral bucket was unit variance scaled. Resonances were assigned by reference to literature values and the Human Metabolome Database and further confirmed by inspection of the 2D and 1D total correlation spectroscopy (TOCSY) and spiking of known compounds.

***Limulus Amebocyte Lysate (LAL) Assay for Endotoxin Quantitation.*** All equipment used was sterile and pyrogen-free. Under sterile conditions, brain tissue was homogenised in 5 volumes of PBS with a 25G needle and 1mL syringe, and diluted 1:40. Quantitation of endotoxin was then performed using the Pierce™ LAL Chromogenic Endotoxin Quantitation Kit (ThermoFisher, UK), as per the manufacturer’s instructions. Briefly, 50uL samples and standards were added to a 96-well plate (Corning Incorporated, USA), equilibrated at 37 °C. After 5 min of incubation, 50uL of LAL enzyme was added, the plate was placed on a shaker for 10 s, and then incubated for a further 10 min. 100uL substrate was then added, the plate shaken, and after exactly 6 min of incubation, 50uL of sterile 25% acetic acid was added to all wells. The plate was shaken again, and absorbance of each well was measured at 405 nm using a CLARIOstar® microplate reader (BMG Labtech Ltd, UK). The read-out from a PBS-only blank was subtracted from all sample wells, and H2O-only was used as the blank for the standards. All samples and standards were assayed in duplicate and the mean taken. The standard curve was generated using a linear regression model in Prism 8 software (Graphpad, USA) and the concentrations of unknown samples were interpolated. Values were expressed as fold change over saline.

***Statistical analysis.*** Data was collected across 3 cohorts of animals; results were combined, and batch effect removal was applied using R software v3.6.1; a linear model of each group was generated and the variation due to batch differences was removed. All statistical analysis was carried out on GraphPad Prism 8.0 software and results presented as mean ± SEM. A p-value < 0.05 was considered significant. One-way ANOVA was performed on all qPCR and endotoxin quantitation, data followed by a Tukey’s post hoc test.

For the analysis of the NMR data, bucket integrals were imported into R software (R foundation for statistical computing, Vienna, Austria) and principle component analysis (PCA), performed using the ropls package, was then used to visualise the degree of separation between the different treatment groups. Metabolites driving the variation between groups were identified by inspection of the PCA loadings. Significant differences in the level of identified metabolites between animals were tested using a one-way ANOVA followed by a Tukey’s post hoc test. Equality of group variances was assumed and tested using the Brown-Forsythe method. If this assumption was not met, Brown-Forsythe and Welch ANOVAs were performed instead, followed by Dunnett T3 post hoc multiple comparisons.

## Results

**Fig. 1:**
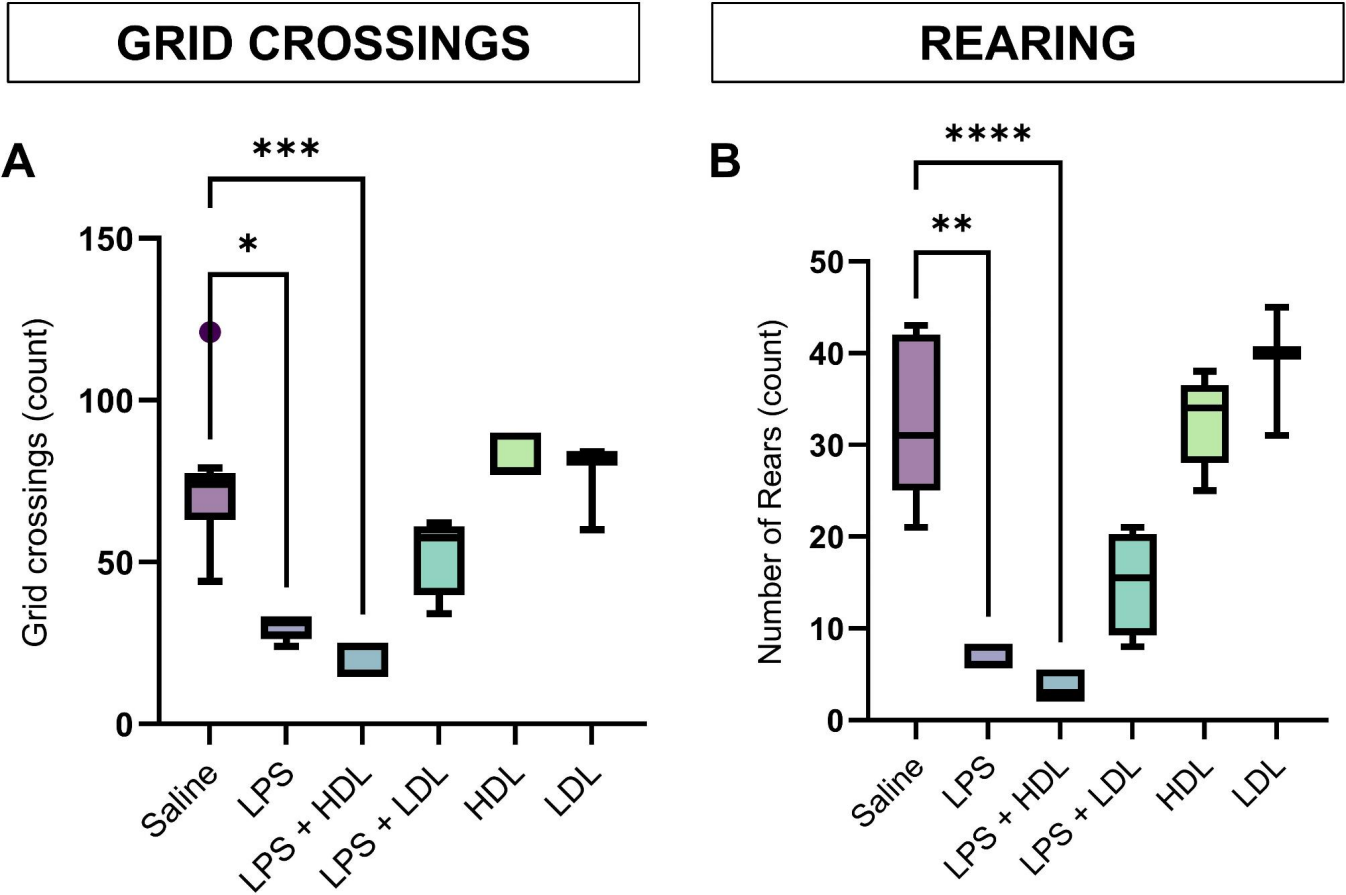
**LPS + LDL but not LPS + HDL ameliorates LPS-induced sickness behaviour.** Adult male C57BL/6 mice received an intraperitoneal injection of either saline (n = 9), LPS (0.5 mg/kg, n = 11) or LPS mixed with lipoprotein immediately prior to administration (HDL, n = 6, 20 mg/kg; LDL, n = 5, 20 mg/kg). 6 h post-injection, animals completed an open field behavioural test, and grid crossings (**A**) and number of rears (**B**) were recorded. Data are presented as Tukey boxplots with Sidak’s test for multiple comparisons, *p < 0.05, **p < 0.01, ***p < 0.001, ****p < 0.0001

***Association of LPS with LDL, but not HDL, ameliorates inflammation-induced sickness behaviour.*** To determine the effect of peripheral HDL and LDL on LPS-induced sickness behaviour, animals underwent an open field paradigm 6 h post-injection (Fig. 1). LPS alone induced a significant decrease in both grid crossings (Saline 74.2 ± 6.9, LPS 29.7 ± 1.1, p < 0.0001) and number of rears (Saline 32.7 ± 2.9, LPS 6.7 ± 1.2; p < 0.0001), indicative of sickness behaviour. When LPS was administered in combination with HDL there was no significant difference in grid crossings (Figs. 1A and 21.1 ± 1.1) or number of rears (Figs. 1B and 3.5 ± 0.7) although a decreasing trend was observed compared to the LPS alone group (p = 0.52 and p = 0.65 respectively). In contrast, when LPS was administered in combination with LDL, animals exhibited a significant increase in grid crossings (Figs. 1A and 52.8 ± 6.3; p < 0.05) compared to LPS alone, as well as a numeric increase in the number of rears (Figs. 1B and 15.0 ± 2.9; p = 0.09). Neither HDL nor LDL alone had any effect on the exploratory behaviour of the animals (HDL alone: grid crossings 81.7 ± 2.6, rears 32.7 ± 2.0; LDL alone: grid crossings 75.3 ± 7.7, rears 38.7 ± 4.1; Fig. 1).

**Fig. 2:**
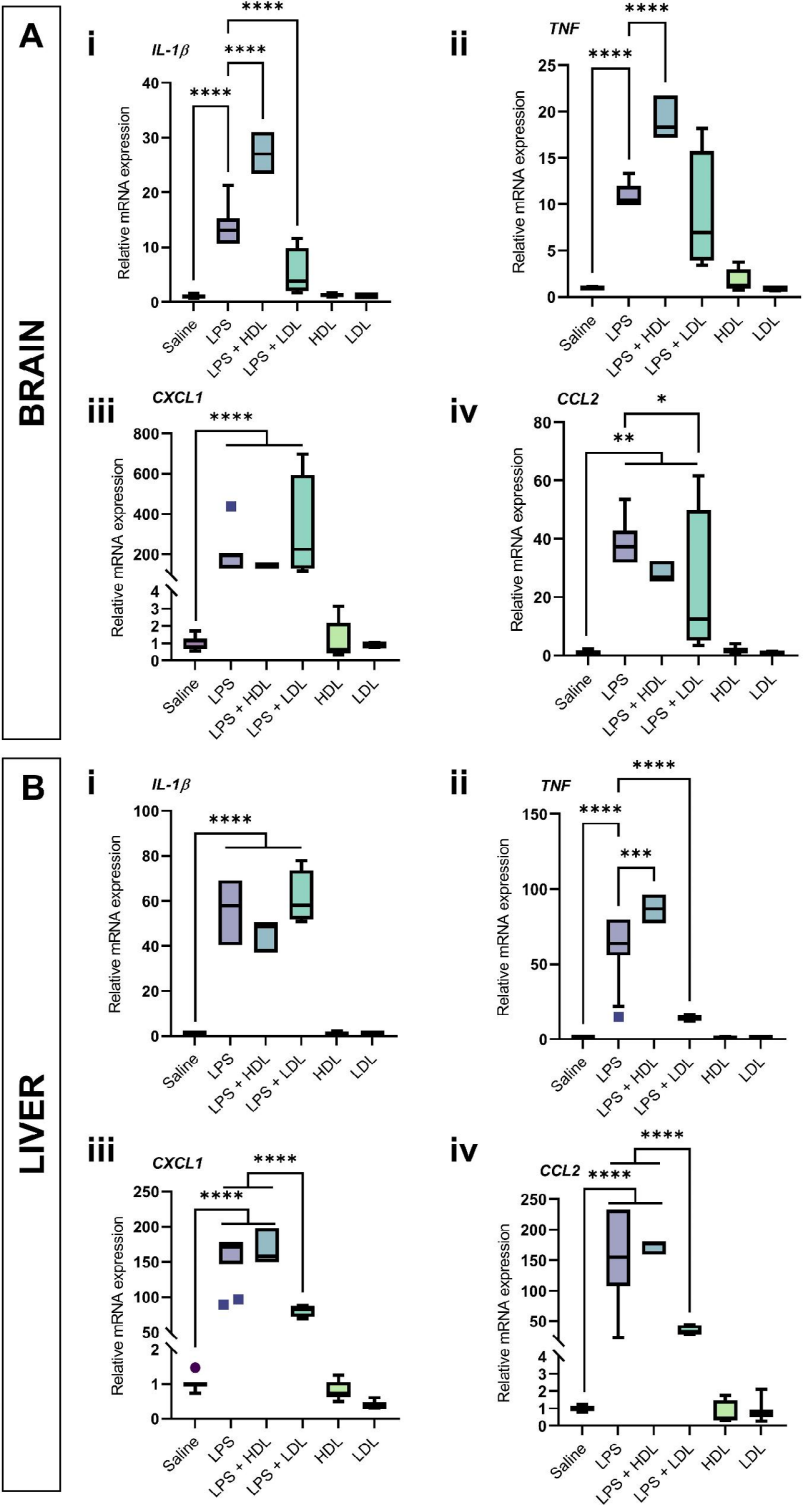
**Expression of inflammation-associated genes in the brain and liver was differentially affected by premixing LPS with HDL and LDL.** Adult male C57BL/6 mice received an intraperitoneal injection of either saline (n = 10), LPS (0.5 mg/kg, n = 11) or LPS mixed with lipoprotein immediately prior to administration (HDL, n = 6, 20 mg/kg; LDL, n = 5, 20 mg/kg). Pro-inflammatory gene expression in the brain (**A**) and liver (**B**) was determined by qPCR. Relative expression of *IL-1β* (i), *TNF* (ii), *CXCL1* (iii) and *CCL2* (iv) was evaluated. Data are presented as Tukey boxplots with Sidak’s test for multiple comparisons, *p < 0.05 **p < 0.01 ***p < 0.001 ****p < 0.0001

***Association of LPS with HDL increases inflammatory cytokine expression in both liver and brain.*** We investigated this behavioural result futher by measuring the expression of pro-inflammatory genes in liver and brain (Fig. 2). Peripheral LPS treatment resulted in significant increases in inflammatory cytokines in the liver and the brain. Combining the administration of LPS with HDL significantly increased PFC expression of *IL-1β* (Fig. 2Ai; LPS 13.8 ± 1.0, LPS + HDL mixed 27.14 ± 1.4; p < 0.0001) and *TNF* (Fig. 2Aii; LPS 11.05 ± 0.4, LPS + HDL mixed 19.07 ± 0.9; p < 0.0001) compared to LPS alone animals, while *CXCL1* and *CCL2* expression were unchanged (Fig. 2Aiii; LPS 202.1 ± 28.3, LPS + HDL 140.4 ± 6.1; Fig. 2Aiv; LPS 39.0 ± 2.2, LPS + HDL mixed 28.3 ± 1.4). Hepatic expression of *TNF* was significantly increased in animals that received the combination of LPS and HDL compared to LPS only animals (Fig. 2Bii; LPS 57.7 ± 6.6, LPS + HDL mixed 86.9 ± 3.5; p < 0.01). However, no significant differences in *IL-1β* (Fig. 2Bi; LPS 55.41 ± 3.5, LPS + HDL mixed 45.5 ± 2.7; p = 0.07), *CXCL1* (Fig. 2Biii; LPS 152.3 ± 9.6, LPS + HDL mixed 168.5 ± 9.4; p = 0.49) or *CCL2* (Fig. 2Biv; LPS 140.1 ± 22.3, LPS + HDL 172.5 ± 4.3; p = 0.52) were evident.

***Association of LPS with LDL ameloirates LPS-induced increased cytokine expression in both liver and brain.*** By comparison, the combination of LPS with LDL decreased PFC *IL-1β* 3-fold compared to animals treated with LPS alone (Fig. 2Ai; LPS + LDL mixed 5.2 ± 2.2; p < 0.0001). Although the expression of *TNF* (Fig. 2Aii; LPS + LDL mixed, 8.9 ± 3.3; p = 0.53), *CXCL1* (Fig. 2Aiii; LPS + LDL mixed 315.3 ± 132.1; p = 0.37), and *CCL2* (Fig. 2Aiv; LPS + LDL mixed 22.6 ± 13.2; p = 0.08) remained unchanged. In the liver, the inverse was observed. Expression of *IL-1β* was unaffected by mixing with LDL (Fig. 2Bi; LPS + LDL mixed 61.2 ± 6.0; p = 0.68), whereas expression of *TNF* (Fig. 2Bii; LPS + LDL mixed 14.16 ± 1.1; p < 0.001), *CXCL1* (Fig. 2Biii; LPS + LDL mixed 80.9 ± 4.1; p < 0.0001) and *CCL2* (Fig. 2Biv; LPS + LDL mixed 37.1 ± 4.0; p < 0.01) were all significantly decreased. In all contexts and comparisons, expression of pro-inflammatory genes in the liver and brains of animals treated with lipoprotein alone was not significantly different from the saline only group (Fig. 2). Overall, HDL generally augmented LPS-induced systemic and brain inflammation, while LDL attenuated it.

***LPS induces significant peripheral metabolite perturbations which are furhter modified by HDL and LDL co-administration.*** Next, we sought to investigate how the acute phase response, induced by LPS, modifies peripheral metabolism using an untargeted metabolomic approach. In parallel, the effects of premixing LPS with HDL (LPS + HDL) or LDL (LPS + LDL) and lipoproteins alone (HDL, LDL) were investigated (Table 1). Plasma and liver metabolites were measured by NMR and visualised by principle component analysis (PCA; Figure S2).

**Table 1:** Summary of the key metabolomic changes in the plasma, liver, and brain by LPS and/or lipoprotein treatment group

METABOLITE		MEAN FC RELATIVE TO SALINE CONTROLS					ANOVA P-VALUE
		LPS	LPS + HDL	LPS + LDL	HDL	LDL	
**PLASMA**	VALINE	1.41****	1.29**	1.32***	1.17	0.96	< 0.0001
	ISOLEUCINE	1.35****	1.34****	1.27***	1.17*	1.01	< 0.0001
	GLUTAMATE	1.32****	1.23**	1.21*	0.91	0.91	< 0.0001
	GLUTAMINE	1.21*	1.14*	1.15*	0.92	0.94	< 0.0001
	LYSINE	1.27****	1.14	1.16	0.96	0.91	< 0.0001
	PHENYLALANINE	1.49****	1.37***	1.45****	1.09	1.04	< 0.0001
	MYO-INOSITOL	1.34****	1.23***	1.22**	0.90	0.98	< 0.0001
	ΒCH_2_ LIPOPROTEIN	1.25****	1.18**	1.08	0.88	0.90	< 0.0001
	CITRATE	1.39****	1.33**	1.17	0.85	0.94	< 0.0001
	GLUCOSE	0.61****	0.60****	0.78	0.85	0.79	< 0.0001
	HDL	1.17	1.45****	1.25	1.40****	0.96	< 0.0001
	LDL	1.09	1.35	1.11	1.35	1.13	0.0918
**LIVER**	GLUTAMATE	1.80****	1.77****	1.78****	1.02	0.89	< 0.0001
	GLUTAMINE	1.40***	1.69****	1.29	0.94	0.76	< 0.0001
	N-ACETYLCYSTEINE	1.33*	1.37*	1.19	1.16	0.79	0.0031
	SUCCINATE	1.29*	1.41***	1.25	1.06	0.93	< 0.0001
	ACETATE	1.23*	1.19	1.23	0.99	0.94	0.0052
	GLUCOSE	0.56***	0.44***	0.57**	0.90	1.21	< 0.0001
	TAURINE	0.67**	0.51****	0.74	0.79	1.12	< 0.0001
**BRAIN**	LACTATE	0.88***	0.82****	0.84***	0.87**	0.88	< 0.0001
	ADENOSINE MONOPHOSPHATE	1.40***	1.34*	1.25	1.15	1.01	0.0008
	ASPARTATE	0.84	0.77*	0.72*	0.74*	0.93	0.0157
	VALINE	1.13	1.17*	1.20*	1.12	1.11	0.0251
	NAD	0.87	0.52*	0.78	0.74	0.60	0.0500
	CITRATE	1.02	1.01	1.07*	1.00	1.09**	0.0016
	LPS (ENDOTOXIN)	1.04	1.20****	1.12**	1.01	0.99	< 0.0001
	HDL	1.16	1.25	1.29	1.28	1.29	0.1302
	LDL	1.10	1.14	1.18	1.19	1.22	0.1299

*P < 0.05, **P < 0.01, ***P < 0.001, ****P < 0.0001, Šídák’s multiple comparisons test

**Fig. 3:**
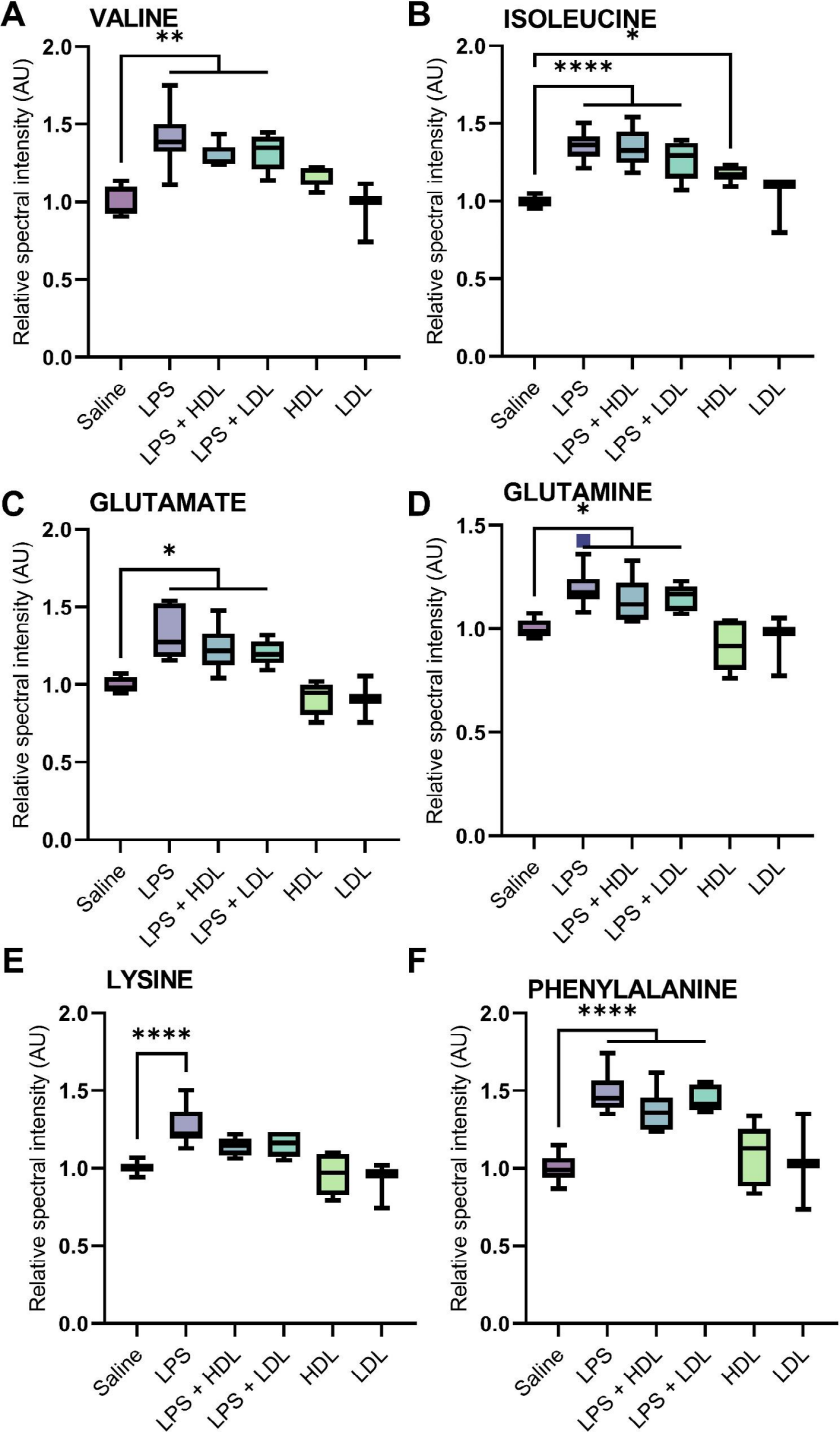
**LPS perturbed plasma metabolites which were not recovered with lipoprotein co-administration.** Adult male C57BL/6 mice received an intraperitoneal injection of either saline (n = 9), LPS (0.5 mg/kg, n = 11) or LPS mixed with lipoprotein immediately prior to administration (HDL, n = 6, 20 mg/kg; LDL, n = 5, 20 mg/kg). Metabolites driving differences between treatment groups by principal component analysis were evaluated. Relative amounts of valine (**A**), isoleucine (**B**), glutamate (**C**), glutamine (**D**), lysine (**E**) and phenylalanine (**F**) were determined. Data are presented as Tukey boxplots analysed by one-way ANOVA with Sidak’s test for multiple comparisons, *p < 0.05, **p < 0.01, ****p < 0.0001

**Fig. 4:**
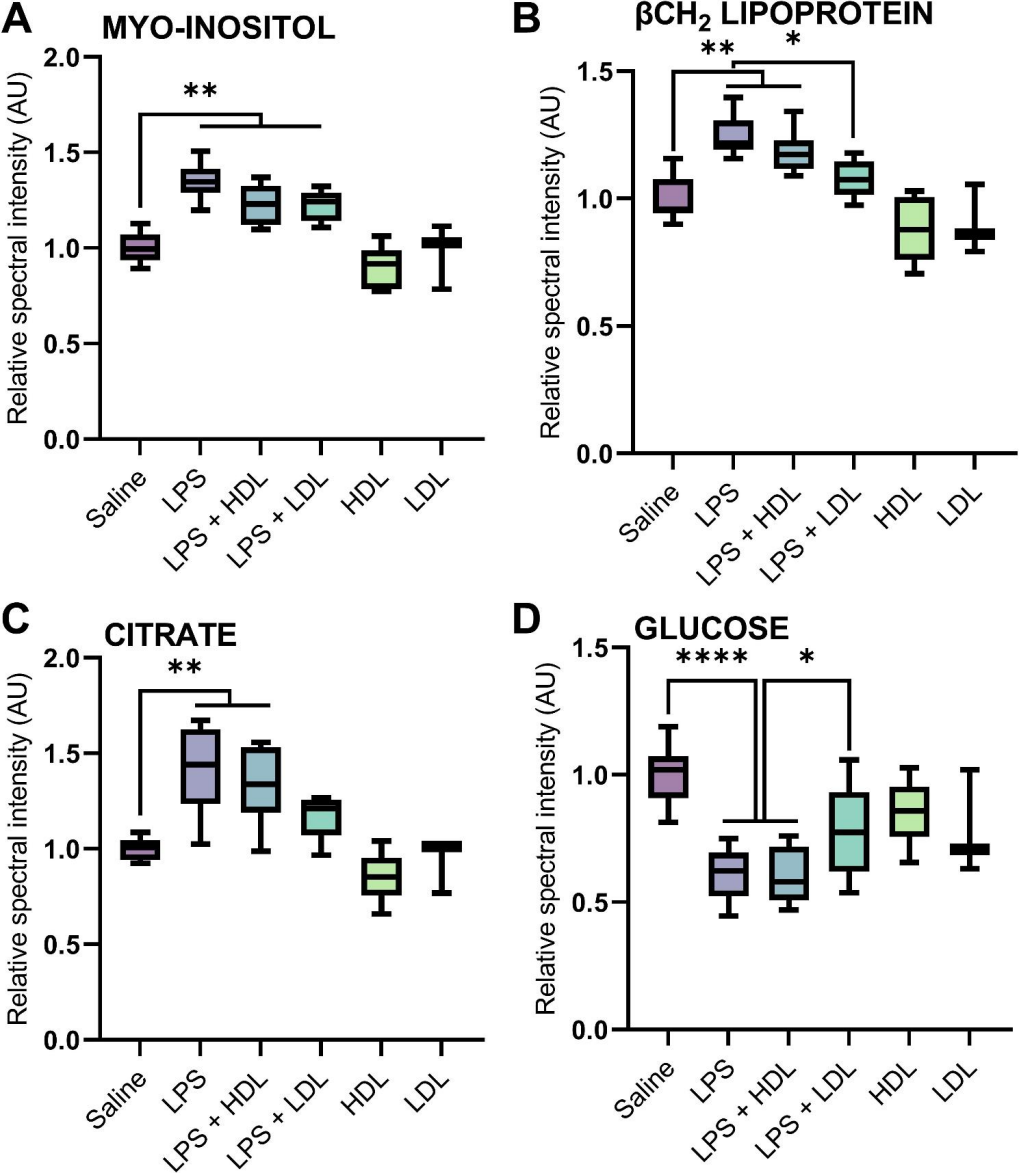
**LPS perturbed plasma metabolites which were partially recovered with LDL co-administration.** Adult male C57BL/6 mice received an intraperitoneal injection of either saline (n = 9), LPS (0.5 mg/kg, n = 11) or LPS mixed with lipoprotein immediately prior to administration (HDL, n = 6, 20 mg/kg; LDL, n = 5, 20 mg/kg). Metabolites driving differences between treatment groups by principal component analysis were evaluated. Relative amounts of myo-inositol (**A**), bCH2 lipoprotein (**B**), citrate (**C**), and glucose (**D**) were determined. Data are presented as Tukey boxplots analysed by one-way ANOVA with Sidak’s test for multiple comparisons, *p < 0.05, **p < 0.01, ****p < 0.0001

In the plasma, PCA revealed that the most significant variation in the data was a result of LPS treatment, indicating that substantial changes to the plasma metabolome occurred as a result of LPS-induced inflammation (Figure S2A). Investigation of the metabolites driving this variation revealed an LPS-induced increase in plasma amino acids (Fig. 3), myo-inositol, fatty acids, and citrate coupled with a signifiacnt decrease in glucose concentration (Fig. 4). Animals treated with LPS alone showed significantly elevated circulating branched chain amino acids (BCAA) valine (p < 0.0001) and isoleucine (p < 0.0001) compared to saline, as well as the amino acids glutamate (p < 0.0001) and glutamine (p < 0.0001), lysine (p < 0.0001) and phenylalanine (p < 0.0001; Fig. 3A-F, respectively).

Other small-molecule energy metabolites and related signalling molecules were also altered by LPS administration (Fig. 4). Plasma myo-inositol (p < 0.0001), bCH2 (p < 0.0001), and citrate (p < 0.0001) levels were increased as a result of LPS treatment (Fig. 4A-C, respectively). On the other hand, glucose (p < 0.0001) was greatly reduced by LPS administration (Fig. 4D).

**Fig. 5:**
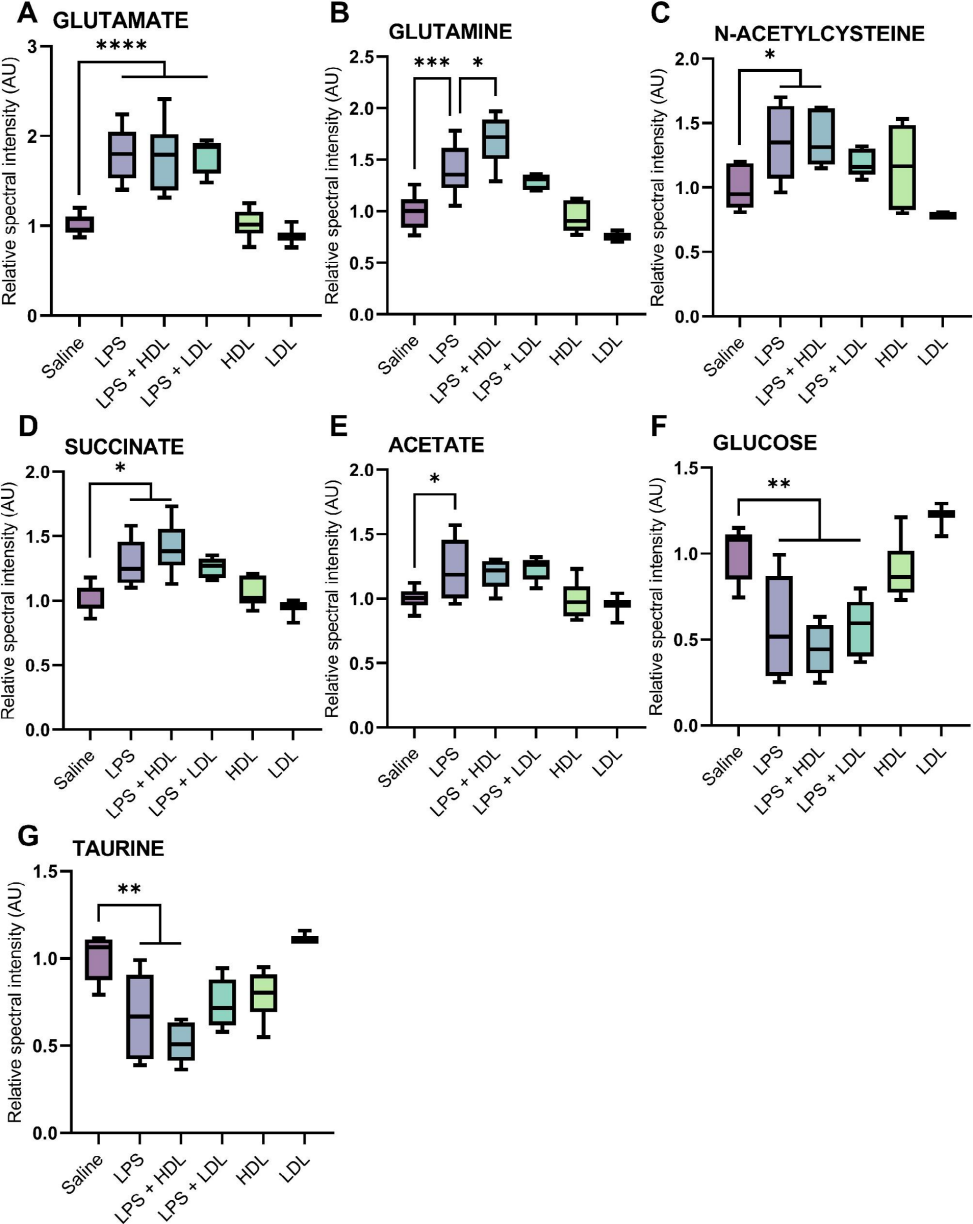
**LPS perturbs liver metabolites which are partially recovered by LDL co-administration.** Adult male C57BL/6 mice received an intraperitoneal injection of either saline (n = 9), LPS (0.5 mg/kg, n = 11) or LPS mixed with lipoprotein immediately prior to administration (HDL, n = 6, 20 mg/kg; LDL, n = 5, 20 mg/kg). Metabolites driving differences between treatment groups by principal component analysis were evaluated. Relative amounts of glutamate (**A**), glutamine (**B**), N-acetylcysteine (**C**), succinate (**D**), acetate (**E**), glucose (**F**) and taurine (**G**) were determined. Data are presented as Tukey boxplots analysed by one-way ANOVA with Sidak’s test for multiple comparisons, *p < 0.05 **p < 0.01 ***p < 0.001 ****p < 0.0001

Multivariate analysis of the liver metabolite data was consistent with that of plasma, in that a large portion of sample variation (separation along the first principle component) could also be explained by LPS treatment (Figure S2B). Consistent with plasma, changes in energy and amino acid metabolism driven by LPS-mediated inflammation (Fig. 5) were obseved in liver. LPS increased the levels of several amino acids, including glutamate (p < 0.0001), glutamine (p < 0.01), and N-acetylcysteine (p < 0.05; Fig. 5A-C, respectively). Succinate (Fig. 5D, p < 0.01) and acetate (Fig. 5E, p < 0.1) were also elevated, though the increase in acetate levels was not statistically significant due to inter-sample variation between LPS animals (Figure S4B). Lastly, glucose (p < 0.01) and taurine (p = 0.01) were depleted in liver tissue as a consequence of LPS treatment (Fig. 5F and G, respectively).

The association of LPS with HDL and LDL produced distinct effects on peripheral metabolism. Whereas the association of LPS with HDL had largely identical effects to LPS alone, the addition of LDL to LPS attenuated some of the observed metabolic changes. The association of LPS with LDL attenuated the LPS-mediated increase in plasma citrate (Fig. 4C). The level of plasma glucose was also significantly greater in LPS + LDL animals compared to those of LPS (p < 0.05) and LPS + HDL (p < 0.05) groups. In the liver, the LPS-mediated increase in N-acetylcysteine (Fig. 5C) and decrease in taurine (Fig. 5G) were also reversed to some extent by premixing with LDL, but not HDL. In contrast, glucose levels were not rescued by LDL association with LPS in the liver (Fig. 5F, p < 0.05 compared to saline) in the same manner observed in plasma.

It should be noted that no overt effects on the peripheral metabolites associated with LPS-induced inflammation were observed as a result of either HDL or LDL alone. In the plasma, HDL increased the level of BCAA isoleucine (p < 0.01) while LDL alone had no effect on blood metabolite levels compared to saline (Fig. 3B). In the liver, taurine levels were significantly higher in LDL-treated animals compared to HDL-treated animals (p < 0.01), though neither were significantly different from saline (Fig. 5G). No other effects of lipoprotein administration on liver metabolites were observed compared to saline controls (Fig. 5).

**Fig. 6:**
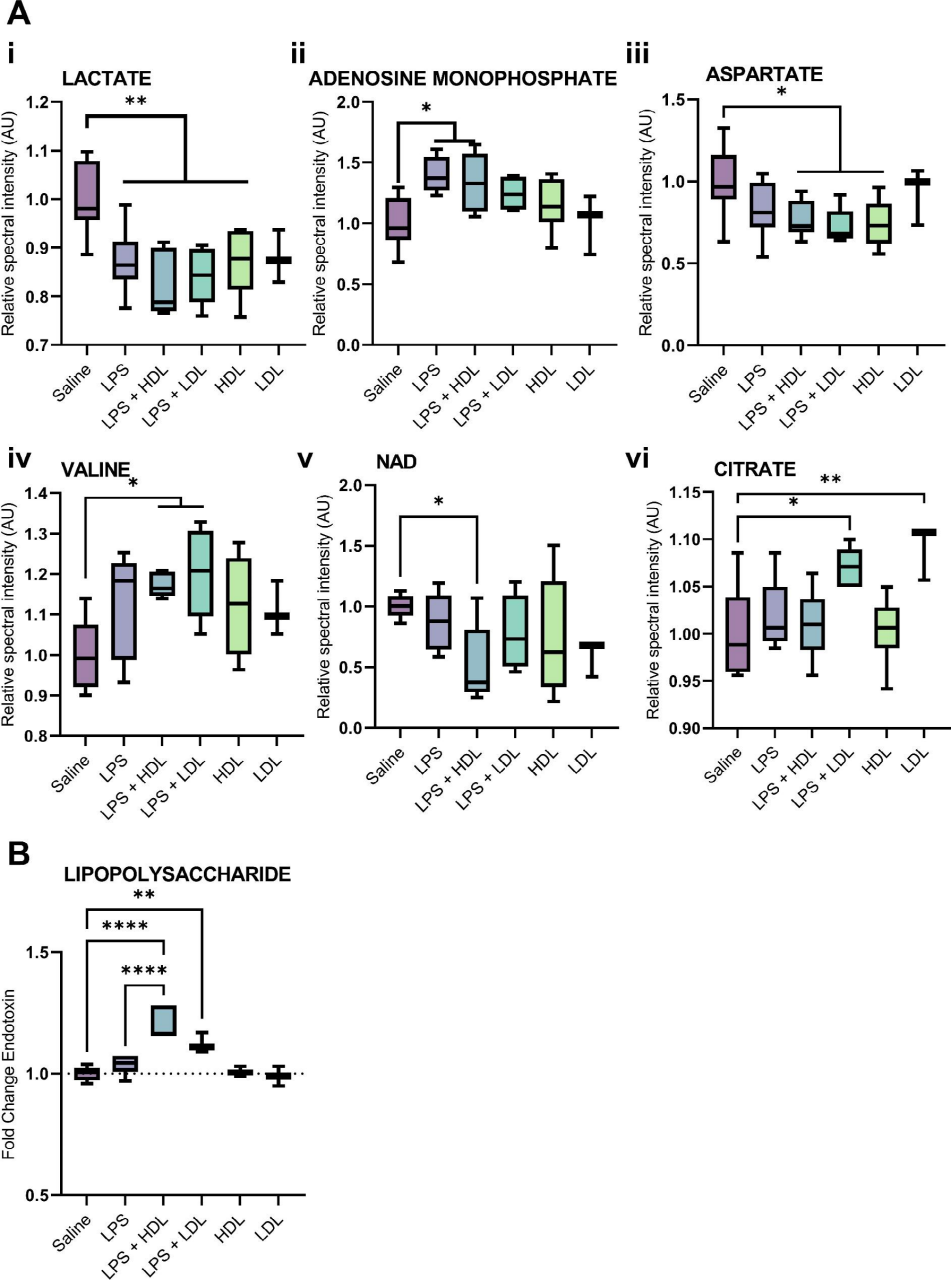
**Peripherally administered LPS in combination with HDL has distinct effects on the brain metabolome and significantly increases the level of brain endotoxin.** Adult male C57BL/6 mice received an intraperitoneal injection of either saline (n = 9), LPS (0.5 mg/kg, n = 11) or LPS mixed with lipoprotein immediately prior to administration (HDL, n = 6, 20 mg/kg; LDL, n = 5, 20 mg/kg). Metabolites driving differences between treatment groups by principal component analysis were evaluated. Relative amounts of lactate (**Ai**), adenosine monophosphate (**Aii**), aspartate (**Aiii**), valine (**Aiv**), NAD (**Av**) and citrate (**Avi**) were determined. Brain lysates were analysed for the presence of endotoxin using a limulus amoebocyte lysate assay (**B**). Data are presented as Tukey boxplots analysed by one-way ANOVA with Sidak’s test for multiple comparisons, *p < 0.05, **p < 0.01, ****p < 0.0001

***Peripherally administered LPS, HDL, and LDL result in perturbations to the brain metabolome.*** The brain metabolic signature of mice treated with LPS in combination with lipoprotein was distinct from saline controls (Figure S2C) resulting in significantly reduced lactate levels (Fig. 6Ai, p < 0.001) and increased levels of adenosine monophosphate (AMP; Fig. 6Aii, p < 0.001).

The administration of either lipoprotein (HDL, p < 0.0001 or LDL, p < 0.001) had no impact on the LPS-induced lactate decrease observed. Admininstration of HDL significantly reduced brain lactate levels compared to saline (Fig. 6Ai) in the absence of LPS. Co-administration of LPS with HDL resulted in signifacntly reduced levels of nicotinamide adenine dinucleotide (NAD) (Fig. 6Av) although no impact of HDL alone on NAD concentration was observed (Fig. 6Av). While LPS resulted in minimal changes to the brain metabolome, several lipoprotein effects (independent of LPS) were observed. Both HDL and LDL resulted in significantly increased valine and decresed aspartate levels in the brain, while LDL alone increased citrate levels (Fig. 6Avi, p < 0.01).

***HDL shuttles LPS to the brain.*** We next aimed to elucidate by which mechanisms HDL mediates increased brain inflammation when pre-mixed with LPS. As HDL binds directly with LPS [6], we hypothesised that the lipoprotein was shuttling LPS directly to the brain. To determine this, a Limulus Amebocyte Lysate (LAL) assay was performed to measure the concentration of endotoxin in brain lysate (Fig. 6B). There was no significant difference between saline and LPS controls (saline 1.0 ± 0.01, LPS 1.0 ± 0.02; p = 0.53). When LPS was pre-mixed with HDL, endotoxin fold change (LPS + HDL mixed 1.2 ± 0.02) was significantly increased when compared with both saline (p < 0.0001) and LPS (p < 0.001). Endotoxin fold change in mice after treatment of LPS + LDL was significantly increased compared to that of mice after saline (LPS + LDL mixed 1.1 ± 0.02; p < 0.05) treatment, but not compared to LPS (p = 0.12).

## Discussion

In this study we observed distinct inflammatory effects of HDL and LDL when co-administered with LPS, and describe marked peripheral metabolic changes associated with the acute phase response. We have shown that the coadministration of human HDL with LPS exacerbates brain markers of neuroinflammation in mice. By contrast, human LDL seemed to prevent LPS-induced inflammation in both the brain and liver. The behavioural tests showed that a mixed treatment LDL + LPS significantly reduced sickness behaviour compared to LPS treatment. The augmentation of LPS-induced brain inflammation in mice may be due to the functional capacity of HDL to bind LPS, and, through metabolic changes associated with the acute phase response, exert a pro-inflammatory effect on tissues distal to the liver. Indeed, LPS + HDL resulted in significantly increased levels of observable endotoxin in the brain while the levels observed in LPS + LDL animals were no different from LPS alone.

The APR is a well-orchestrated series of systemic physiological processes occurring at the onset of the innate immune response. Bacterial infections typically evoke a strong APR. LPS, a gram-negative bacterial endotoxin known to induce inflammation [42], can directly activate innate immune cells such as tissue macrophages, blood monocytes, and neutrophils [3]. This leads to the local induction of key pro-inflammatory cytokines, TNF and IL-1ß [44, 51], and the profound modulation of protein synthesis by hepatocytes in the liver [10]. These hepatic alterations translate to substantial changes in HDL protein composition, most notably with ApoA-I being displaced from HDL by the incorporation of SAA and LBP [52].

To determine whether the distinct behavioural effects of LPS in combination with HDL or LDL administration were due to underlying differences in brain or peripheral inflammation, we investigated the gene expression of key cytokines involved in the acute phase response, including *IL-1ß, TNF, CXCL1*, and *CCL2*. In the brain, LPS treatment led to a robust increase in the expression of all pro-inflammatory cytokines. The co-administration of HDL augmented this inflammatory response, significantly elevating expression of *IL-1ß* and *TNF*, though *CCL2* expression was reduced (Fig. 2A). IL-1ß and TNF are two of the most sensitive cytokines associated with TLR4 signalling, so any increased inflammation due to HDL co-administration is likely to be discerned through increased transcription of these inflammatory mediators if not others [41]. Peripherally, premixing LPS with HDL also tended to exacerbate the inflammatory response, with a significant increase in *TNF* gene expression in the liver (Fig. 2). Importantly, the administration of human HDL or LDL alone did not elicit a pro-inflammatory immune response (Fig. 2).

Given the increase in central inflammation in the HDL cohorts, we hypothesised that HDL, but not LDL, may be shuttling bound LPS to the brain. To investigate this, we conducted an endotoxin assay on brain homogenates. Whilst mice that received an intraperitoneal injection of saline only or LPS only had equivalently low levels of endotoxin, mice that were injected with a premixed solution of LPS and HDL showed significantly increased levels of brain endotoxin (Fig. 6B). This may suggest that HDL, in an inflammatory environment, is able to transport LPS to the brain. It should be noted that we could not distinguish between the detection of endotoxin in brain vasculature from brain parenchyma, and thus whether HDL is able to transport LPS across the blood brain barrier and directly stimulate parenchymal macrophages, though this has been previously demonstrated [58].

In contrast to the effect of HDL on LPS-induced inflammation, LDL attenuated both central and peripheral pro-inflammatory gene expression (Fig. 2). As LDL may disperse its cargo away from the liver toward other cells, predominately through the interaction between ApoB-100 and LDL receptors [59], this observation may be due to the ability of LDL to scavenge LPS without overtly activating proximal innate immune cells. Oxidation of administered LDL is unlikely to contribute to the effects observed in this study. Our results are somewhat counterintuitive because the inflammatory properties of modified LDLs have been a principal focus of atherosclerosis research for decades where they contribute to inflammation in the arterial wall [47]. For example, modified LDLs, such as Oxidized LDL (oxLDL) and minimally modified LDL (mmLDL), are recognized by the scavenger receptor, and which mediates the uptake and formation of lipid-loaded foam cells that are typical of atherosclerotic lesions. It is known that LDL maybe modified in the arterial intima, and more slowly in plasma circulation. However, they have been detected in blood only at a very low concentration, which is strongly antioxidant enriched [13]. Fully oxLDL are reportedly quickly cleared from circulating blood mainly by hepatic Kupffer cells [32]. Indeed, the Kupffer cells removed > 90% or injected modified LDLs in 5 min. Thus it is unlikely that there is to be a contribution from modified LDLs in our experiments in the acute timeframe.

In addition, oxLDL reduces cytokine production after activation of plasmacytoid dendritic cells in vitro, suggesting that this may be one potential mechanism by which administered LDL may be anti-inflammatory in the presence of endotoxin [18]. More specific to this study, pre-treatment of LPS-stimulated macrophages with oxLDL reduced mRNA expression of the pro-inflammatory cytokine *IL-6* and increased the expression of the anti-inflammatory cytokine *IL-10* via interacting with macrophage scavenger receptors [48]. Thus, premixing LPS and LDL may partially reduce the extent of the acute phase response after LPS-induced inflammation. Further experiments are required to clarify the precise mechanisms by which HDL and LDL interact with LPS to modulate the immune response.

Consistent with the findings from qPCR, acute peripheral LPS administration also showed marked changes on peripheral metabolism that reflect altered energy requirements during the APR. One of the key changes in the plasma metabolome was the effect of LPS on increasing the level of plasma BCAAs (Fig. 3A and B). BCAA elevation may be downstream from LPS-mediated immune activation, as BCAAs are known to have pro-inflammatory effects via mTORC1 [62]. Other amino acids in plasma were elevated by LPS treatment, including glutamate, glutamine, lysine, and phenylalanine (Fig. 3 C-F). When stimulated, immune cells greatly increase their metabolic demand [55]. Consequently, in proliferating immune cells amino acids are preserved at the expense of glucose, as glycolysis becomes the key form of energy metabolism [2]. This switch to glycolysis is particularly pronounced in LPS-stimulated organisms via the activation of TLR4, known as the Warburg effect [49]. As a result, plasma glucose levels were significantly depleted (Fig. 4D). Like in plasma, liver glucose was also depleted in LPS-treated animals (Fig. 5F). Glutamate (Fig. 5A), glutamine (Fig. 5B), and acetate (Fig. 5E) were increased by LPS treatment relative to saline and may function as precursors for immune-related anabolic processes.

Peripheral metabolism was also differentially affected by the association of LPS with HDL or LDL. In line with the findings from behavioural and gene expression analysis, LDL attenuated the LPS-induced changes in glucose, citrate, and taurine (Fig. 4) while LPS + HDL tended to exacerbate those changes. In the liver, glutamine levels were significantly higher in LPS + HDL animals compared to LPS and LPS + HDL animals. Given the central role glutamine plays in the metabolic reprogramming of the innate immune response [5, 35, 36], this may reflect the increased liver inflammation in LPS + HDL treated animals as demonstrated by gene expression analysis of pro-inflammatory cytokines (Fig. 2).

In the brain, the effect of peripheral LPS administration on metabolism was less pronounced than that observed in either blood or liver suggesting that, while coadministration of lipoproteins resulted in increased endotoxin in the brain, the behavioural changes observed are largely a result of modulation of the peripheral immune response and metabolic pathways. The limited number of metabolic changes observed in the brain is somewhat expected as peripheral administration of LPS will produce a predominantly peripheral immune response although the use of whole brain lysates for metabolomics analysis may have also played a role; the heterogeneity of the tissue may dampen region-specific group differences in comparison to the more homogenous liver.

Interestingly, a significant reduction in brain NAD concentration were only observed when LPS was co-administered with (Fig. 6A-v) suggesting that this may be a direct result of increased brain endotoxin levels. NAD has shown to be reduced in both peripheral and brain inflammatory diseases [17, 63], and its levels are directly linked to neuronal function and survival [45]. Further research on the link between endotoxemia and neuroinflammation should investigate the link between NAD levels and the resolution of inflammatory activity.

Citrate was significantly elevated in the brain of LDL-treated animals, compared to saline (6Avi). This was irrespective of whether LDL was first premixed with LPS and suggests a unique role of LDL in being able to increase brain citrate levels. Citrate has previously been demonstrated to reduce brain inflammation and oxidative stress in a mouse model of LPS-induced systemic and central inflammation [1]. It is possible that here the LDL-mediated increase in brain citrate protected against LPS-induced brain inflammation, resulting in the reduced levels of brain IL-β and TNF observed, though further experiments are needed to clarify this.

While an abundance of research has demonstrated the involvement of specific HDL-associated proteins with the innate immune system, only recently have the advent of proteomic techniques in mass spectrometry demonstrated the functional capacity of HDL. This extends the general dogma of lipoproteins as vehicles of lipid transport to include complex roles in innate immunity and haemostasis [46]. Indeed, while the protein complement of LDL is dominated by apolipoprotein B (Figure S1E), a heterogeneous assortment of proteins have been described as constituents of HDL [52]. Under infectious and inflammatory conditions that elicit the APR, HDL undergoes significant remodelling and increased pro-inflammatory activity [57]. Recent research indicates that this is due in part to the association of SAA with HDL during infection [19]. In both humans and mice, SAA is one of the major acute phase proteins (APPs), in which synthesis can be upregulated > 1000-fold during the APR [10]. SAA is the primary apolipoprotein associated with HDL during the APR [8]. The ability of SAA to transiently but dramatically increase the levels of several cytokines, including IL-1ß, CCL2, and TNF through the stimulation of monocytes and macrophages has also been demonstrated [54].

Increased endotoxin levels in the brain likely explain the pro-inflammatory metabolite profiles and the increased levels of inflammatory cytokines observed in LPS + HDL treated animals. In contrast, the extent by which LDL modulated endotoxin transport and brain metabolism is unlikely to fully account for the significant amelioration of sickness behaviour and inflammatory cytokines observed. Thus, the anti-inflammatory properties of LDL observed in this study are most likely a result of modification of peripheral metabolic processes and the acute phase response as supported by the metabolic data presented. Lipoproteins – along with other components of the innate immune system – may act as a double-edged sword in endotoxemia and sepsis. Binding LPS may ultimately function to clear LPS from blood and tissues but may also induce an inflammatory response elsewhere. The association of LPS and HDL during infection may contribute to the pro-inflammatory activation of parenchymal macrophages of the CNS contributing to sepsis induced neuroinflammation.

While we confirmed the presence of ApoA-I, and absence of ApoB, on administered HDL particles (Fig. S1E), future work should aim to differentiate further between HDL subclasses and their immunomodulatory effects. Subsequent proteomic and lipidomic analysis of HDL and LDL lipoproteins isolated from mice in the aftermath of the acute phase response could help determine the key components of HDL that contribute to its pro-inflammatory effects. Moreover, although LPS displays greatest affinity for HDL amongst the lipoprotein subclasses [29], there is evidence to suggest that HDL subsequently redistributes endotoxin to LDL under the physiological conditions of the acute phase response. This process is complementary to the acute-phase remodelling of HDL and may contribute to its pro-inflammatory effects [30]. Therefore, the rate of exchange of LPS between lipoprotein subclasses when co-administered with HDL or LDL should also be investigated.

We recognise the limitations of this study. While we identified a protective role of LDL, when premixed with LPS, on brain and liver pro-inflammatory gene expression, liver endotoxin levels were not assessed. Subsequent experiments are required to determine the specific mechanisms behind the anti-inflammatory effects of LDL, for example, whether LDL shuttles LPS to the liver for subsequent secretion into the intestines [12]. This may be achieved by utilising a fluorescent labelled lipopolysaccharide conjugate, or isolating LPS + LDL particles from target organs. In addition, while the measurement of cytokine expression by mRNA levels was informative, this unilateral approach to assessing inflammation is a limitation of our study. Complementary protein and histological readouts of the inflammatory response would be a useful addition to future experiments.

It would also be useful to understand how different timepoints modify the relationship between lipoprotein administration and the host inflammatory response to LPS. Indeed, a further limitation of this study is the single dose regime of lipoprotein administration and single timepoint. While the single 6-hour timepoint was determined by our interest in the acute phase response and associated sickness behaviours, an extended timepoint and/or treatment regime may reveal additional roles for modified lipoproteins, for example oxLDL. Only male mice were used in these experiments, and sex differences should be a feature of follow-up studies. Lastly, it should also be noted that the translatability of the results to humans is not yet clear, due to the different lipoprotein profiles between mice and humans.

The results of this study present some possible therapeutic implications. Most notably, elevated levels of HDL, in the presence of LPS, may exacerbate neuroinflammation. Consequently, an ideal therapeutic range of HDL may be advisable, as opposed to simply a recommended minimum blood concentration. This is supported by recent clinical evidence, whereby both low and high HDL levels were associated with significantly increased odds of all-cause and cause-specific mortality [31, 60]. While HDL is also thought to play a protective immunomodulatory role in sepsis, this appears to be via an ApoA-I dependent mechanism [6, 33]. HDL and LDL may be useful targets in modulating the acute phase response, and greater insight into their immunomodulatory roles may help prevent neuroinflammation and subsequent neurodegeneration associated with endotoxemia.

In summary, our results indicate that HDL and LDL play distinct roles during the acute phase of LPS-induced inflammation. LDL but not HDL can ameliorate the LPS-induced sickness behaviour represented by an open field test. Furthermore, we showed HDL can shuttle LPS to the brain to promote neuroinflammation, but LDL has anti-neuroinflammatory properties in this LPS-induced mouse model. This study provides a platform to further explore how lipoproteins and other immunometabolites mediate brain-periphery communication during the acute phase of LPS-induced inflammation.

## Electronic supplementary material

Below is the link to the electronic supplementary material.

**Supplementary Material**: **Figure S1. Relative concentration of HDL and LDL in plasma (A, B) and brain extracts (C, D)**. Confirmation of purity of lipoprotein samples by immunoblot (D). Relative to saline controls, HDL levels in plasma were significantly increased 6hrs after intraperitoneal administration with HDL alone and HDL in combination with LPS (both p < 0.0001). HDL levels in plasma were also significantly higher in HDL+LPS treated mice compared to LPS controls (p = 0.0035). To ensure that lipoproteins used in this study were free of contamination, reagents were stained by immunoblot. VLDL, LDL, HDL, and EV control (TSG101), at 2 different protein concentrations were measured. **Figure S2. Scores plots of multi-variate analysis**. Adult male C57BL/6 mice received an intraperitoneal injection of either saline (n=9), LPS (0.5mg/kg, n=11) or LPS mixed with lipoprotein immediately prior to administration (HDL, n=6, 20mg/kg; LDL, n=5, 20mg/kg). Control animals received an intravenous injection of HDL (n=6) or LDL alone (n=3). Fresh tissue was collected 6 hours post-insult, metabolites extracted and measured using 1H NMR. Scores plots of plasma (A), liver (B) and brain (C) samples were analysed by PCA using R 3.3.2 with the ROPLS package and in-house scripts.

## Data Availability

The datasets used and/or analysed during the current study are available from the corresponding author on reasonable request.
