# Supplementary material for: HDL and LDL have distinct, opposing effects on LPS-induced brain inflammation
Source: Lipids Health Dis. 2023 Apr 24;22:54. doi: 10.1186/s12944-023-01817-z (PMC10124044; doi:10.1186/s12944-023-01817-z)
Supplement: Supplementary file 1 — Supplementary Material: Figure S1. Relative concentration of HDL and LDL in plasma (A, B) and brain extracts (C, D). Confirmation of purity of lipoprotein samples by immunoblot (D). Relative to saline controls, HDL levels in plasma were significantly increased 6hrs after intraperitoneal administration with HDL alone and HDL in combination with LPS (both p < 0.0001). HDL levels in plasma were also significantly higher in HDL+LPS treated mice compared to LPS controls (p = 0.0035). To ensure that lipoproteins used in this study were free of contamination, reagents were stained by immunoblot. VLDL, LDL, HDL, and EV control (TSG101), at 2 different protein concentrations were measured. Figure S2. Scores plots of multi-variate analysis. Adult male C57BL/6 mice received an intraperitoneal injection of either saline (n=9), LPS (0.5mg/kg, n=11) or LPS mixed with lipoprotein immediately prior to administration (HDL, n=6, 20mg/kg; LDL, n=5, 20mg/kg). Control animals received an intravenous injection of HDL (n=6) or LDL alone (n=3). Fresh tissue was collected 6 hours post-insult, metabolites extracted and measured using 1H NMR. Scores plots of plasma (A), liver (B) and brain (C) samples were analysed by PCA using R 3.3.2 with the ROPLS package and in-house scripts. [file 12944_2023_1817_MOESM1_ESM.docx]

**Supplementary Materials**

HDL and LDL have distinct, opposing effects on LPS-induced brain inflammation

**Daniel E. Radford-Smith^1,2^*, Abi G. Yates^1,2^, Laila Rizvi^1^, Daniel C. Anthony^1^ & Fay Probert^2^**

^1^ Department of Pharmacology, Medical Sciences Division, University of Oxford, Oxford, UK

^2^ Department of Chemistry, University of Oxford, Oxford, UK

*Correspondence to: daniel.radford-smith@pharm.ox.ac.uk; Tel.: +44 (0)1865 281135


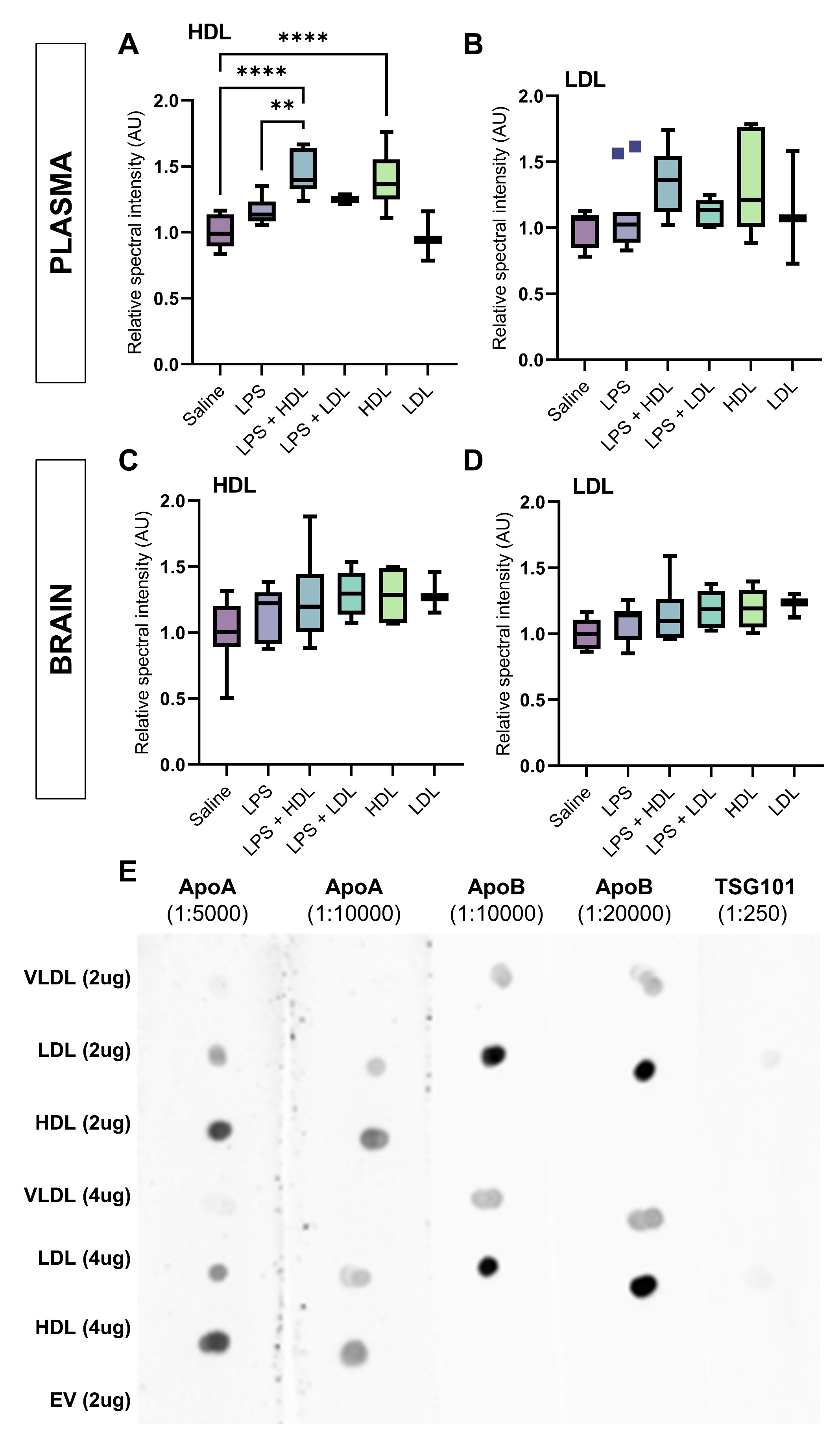


**Figure S1. Relative concentration of HDL and LDL in plasma (A, B) and brain extracts (C, D). Confirmation of purity of lipoprotein samples by immunoblot (D).** Relative to saline controls, HDL levels in plasma were significantly increased 6hrs after intraperitoneal administration with HDL alone and HDL in combination with LPS (both p < 0.0001). HDL levels in plasma were also significantly higher in HDL+LPS treated mice compared to LPS controls (p = 0.0035). To ensure that lipoproteins used in this study were free of contamination, reagents were stained by immunoblot. VLDL, LDL, HDL, and EV control (TSG101), at 2 different protein concentrations were measured.


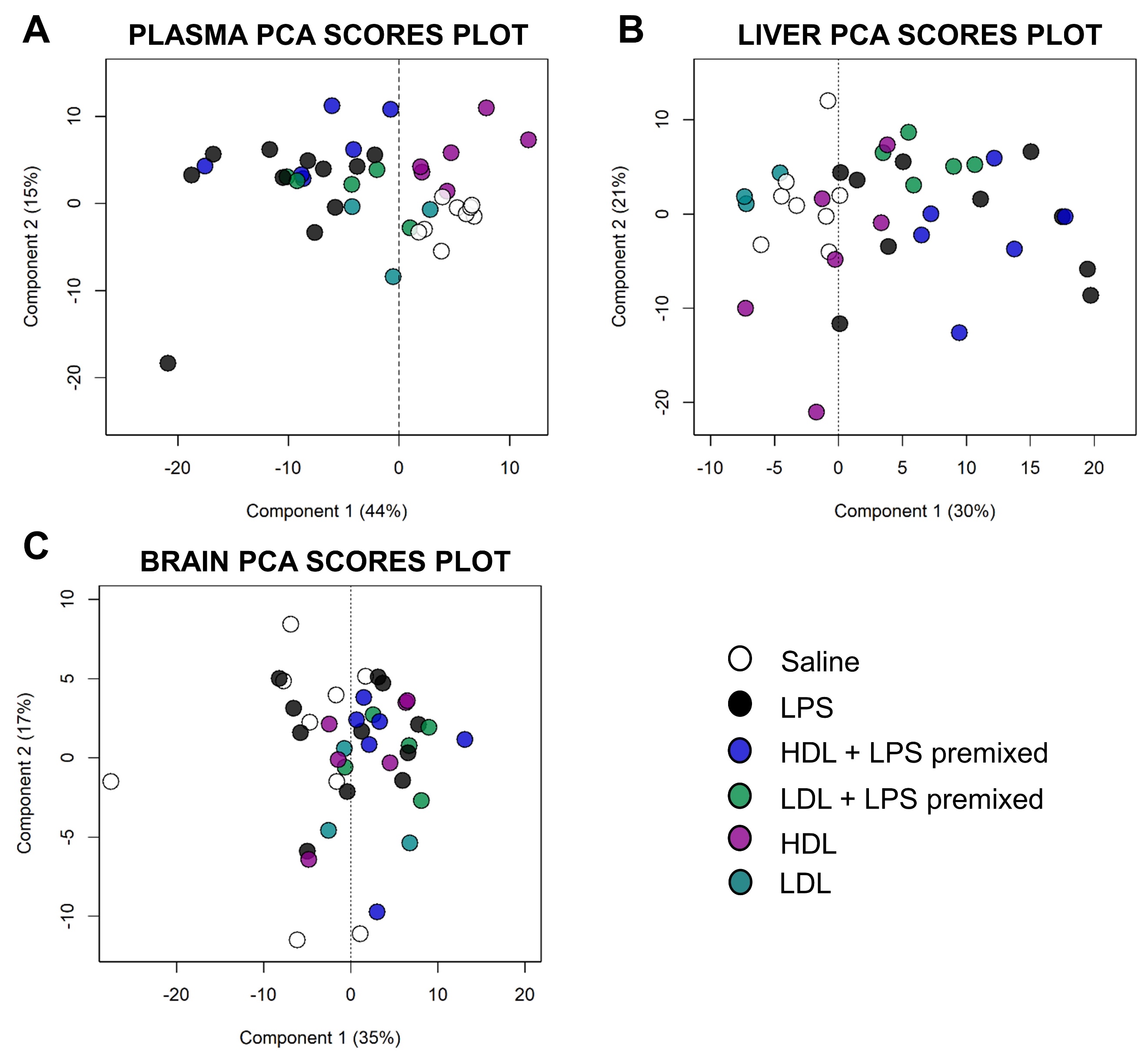


**Figure S2. Scores plots of multi-variate analysis.** Adult male C57BL/6 mice received an intraperitoneal injection of either saline (n=9), LPS (0.5mg/kg, n=11) or LPS mixed with lipoprotein immediately prior to administration (HDL, n=6, 20mg/kg; LDL, n=5, 20mg/kg). Control animals received an intravenous injection of HDL (n=6) or LDL alone (n=3). Fresh tissue was collected 6 hours post-insult, metabolites extracted and measured using ^1^H NMR. Scores plots of plasma (A), liver (B) and brain (C) samples were analysed by PCA using R 3.3.2 with the ROPLS package and in-house scripts.
